# Summarizing Finite Mixture Model with Overlapping Quantification

**DOI:** 10.3390/e23111503

**Published:** 2021-11-13

**Authors:** Shunki Kyoya, Kenji Yamanishi

**Affiliations:** Graduate School of Information Science and Technology, The University of Tokyo, 7-3-1 Hongo, Bunkyo-ku, Tokyo 113-8656, Japan; yamanishi@mist.i.u-tokyo.ac.jp

**Keywords:** model-based clustering, merging mixture components, component overlap, interpretability

## Abstract

Finite mixture models are widely used for modeling and clustering data. When they are used for clustering, they are often interpreted by regarding each component as one cluster. However, this assumption may be invalid when the components overlap. It leads to the issue of analyzing such overlaps to correctly understand the models. The primary purpose of this paper is to establish a theoretical framework for interpreting the overlapping mixture models by estimating how they overlap, using measures of information such as entropy and mutual information. This is achieved by merging components to regard multiple components as one cluster and summarizing the merging results. First, we propose three conditions that any merging criterion should satisfy. Then, we investigate whether several existing merging criteria satisfy the conditions and modify them to fulfill more conditions. Second, we propose a novel concept named clustering summarization to evaluate the merging results. In it, we can quantify how overlapped and biased the clusters are, using mutual information-based criteria. Using artificial and real datasets, we empirically demonstrate that our methods of modifying criteria and summarizing results are effective for understanding the cluster structures. We therefore give a new view of interpretability/explainability for model-based clustering.

## 1. Introduction

### 1.1. Motivation

**Figure 1:**
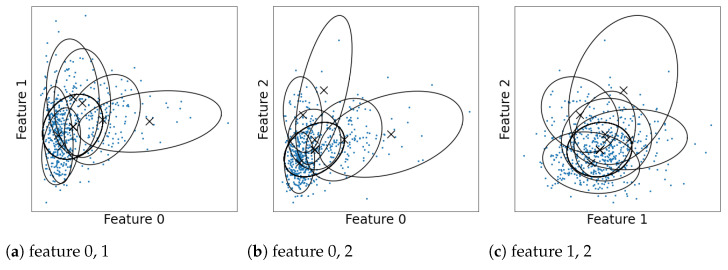
Estimated Gaussian components for the Wisconsin breast cancer dataset [3].

Finite mixture models are widely used for modeling data and finding latent clusters (see McLachlan and Peel [1] and Fraley and Raftery [2] for overviews and references). When they are used for clustering, they are typically interpreted by regarding each component as a single cluster. However, the one-to-one correspondence between the clusters and mixture components does not hold when the components overlap. This is because the clustering structure then becomes more ambiguous and complex. Let us illustrate this using a Gaussian mixture model estimated for the Wisconsin breast cancer dataset in Figure 1 (details of the dataset and estimation are discussed in Section 8.2). A number of the components overlap with one another, which makes it difficult to estimate the shape of distribution or number of clusters. Therefore, we need an analysis of the overlaps to correctly interpret the models.

We address this issue from two aspects. In the first aspect, we consider merging mixture components to regard several components as one cluster. We repeatedly select the most overlapping pairs of components to merge them. In this procedure, it is important how the degree of overlap is measured. A number of criteria for measuring cluster overlaps have been proposed [4,5,6], but they have not yet been compared theoretically. We give a theoretical framework for comparing merging criteria by defining three essential conditions that any method for merging clusters should satisfy. The more conditions any method satisfies, the better it is. From this viewpoint, we evaluate the existing criteria (entropy (Ent) [4], directly estimated misclassification (DEMP) [5] probability, mixture complexity (MC) [7]). We also modify these existing criteria so that they can satisfy more essential conditions.

**Figure 2:**
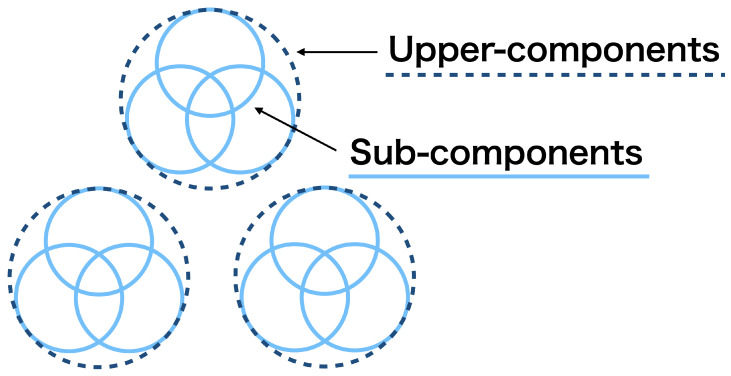
Upper-components and sub-components.

In the second aspect, we consider how to summarize the merging results quantitatively. After merging mixture components, we obtain two types of clustering structures; those among the upper-components and those among sub-components within each upper-component, as illustrated in Figure 2. These structures might be still ambiguous because the upper-components are determined to be the different clusters, but they may overlap; the sub-components are determined to belong to the same cluster, but they may be scattered in the cluster. Therefore, we need to evaluate the degree to which the upper- and sub-components are discriminated as different clusters. We realize this using the notions of *mixture complexity* (MC) [7] and *normalized mixture complexity* (NMC). They give real-valued quantification of the number of effective clusters and the degree of their separation, respectively. We therefore develop a novel method for cluster summarization.

Our hypotheses in this paper are summarized as follows:

- Modifying merging criteria based on essential conditions can improve the ability to find cluster structures in the mixture model.
- Cluster summarization based on MC and NMC effectively describes the clustering structures.

We empirically verify them by experiments, using artificial and real datasets.

### 1.2. Significance and Novelty of This Paper

The significance and novelty of this paper is summarized below.

#### 1.2.1. Proposal of Theoretical Framework for Evaluating Merging Criteria

We give a theoretical framework for evaluating merging methods by defining the *essential conditions*. They are necessary conditions that any merging criterion should satisfy: (1) the criterion should take the best value when the components are entirely overlapped, (2) it should take the worst value when the components are entirely separated, and (3) it should be invariant with respect to the scale of the weights. We empirically confirm that the more essential conditions any merging method satisfies, the better the clustering structure obtained in terms of larger interdistances and smaller intradistances.

#### 1.2.2. Proposal of Quantitative Clustering Summarization

We propose a method for quantitatively summarizing clustering results based on MC and NMC. MC is an extended concept of the number of clusters into a real number from the viewpoint of information theory [7]. It quantifies the diversity among the components, considering their overlap and weight bias. NMC is defined by normalizing MC to remove the effects of weight bias. It quantifies the degree of the scatter of the components based only on their overlap. Furthermore, MC and NMC have desirable properties for clustering summarization: they are scale invariant and can quantify overlaps among more than two components. We empirically demonstrate that our MC-based method effectively summarizes the clustering structures. We therefore give a novel quantification of clustering structures.

## 2. Related Work on Finite Mixture Models and Model-Based Clustering

In this section, we present related work on finite mixture models and model-based clustering in four parts: roles of overlap, model, optimization, and visualization. The overlap has a particular impact on the construction of models.

### 2.1. Roles of Overlap

There has been widespread discussion about the roles of overlap in finite mixture models. One argues that the overlap is emerged to represent various distributions. While this flexibility is beneficial for modeling the data, various issues arise in applying them to clustering. For example, McLachlan and Peel [1] pointed out that some skew clusters required more than one Gaussian component to be represented. Moreover, Biernacki et al. [8] pointed out that the number of mixture components selected for estimating densities was typically more than that of clusters because of overlapping. Model selection methods based on clustering (complete) likelihood, such as the integrated complete likelihood (ICL) [8], the normalized maximum likelihood (NML) [9,10], and the decomposed normalized maximum likelihood (DNML) [11,12], have been proposed to obtain less-overlapping mixtures so that one component corresponds to one cluster. However, they have problems in that they need to define the shape of the clusters in advance. This leads to a trade-off between shape flexibility and component overlap in model-based clustering.

Others argue that the overlap represents that the data belong to more than one cluster. For example, in clustering documents by their topics, the data may have several topics. Such issues have been widely discussed in the field of overlapping clustering. For example, Banerjee et al. [13] extended the mixture model to allow the data to belong to multiple clusters based on membership matrices. Fu and Banerjee [14] considered the product of cluster distributions to represent multiple memberships of the data. Xu et al. [15] proposed methods for describing more complex memberships by calculating correlation weights between the data and the cluster. While these methods allow complex relationships between the data and the clusters, cluster shapes become simple.

The overlap is also used for measuring the complexity of clustering structures in the concept of MC [7]. It is a non-integer valued quantity, which implies the uncertainty of determining the number of clusters. MC was introduced in the scenario of change detection in [7]. This paper gives a new application scenario of MC in the context of quantifying clustering structures. Moreover, this paper also newly introduces NMC as a variant of MC, which turns out to be most effective in this context.

### 2.2. Model

We discuss the issue of constructing models achieving both flexible cluster shapes and interpretability. Allowing each cluster to have complex shapes is a solution to tackle this. For example, mixtures of non-normal distributions have been proposed for this purpose, as reviewed by Lee and McLachlan [16]. Modeling each cluster as a finite mixture model, called the mixture of mixture model or multi-layer mixture model, has been considered in this regard. Various methods have been proposed to estimate such mixture models based on maximum likelihood estimation [17,18] and Bayesian estimation [19,20]. However, additional parameters are required for assigning sub-components to upper-clusters in many cases because changes of assignment do not change the overall distribution. Merging mixture components [4,5,6] is an alternative way of the composition of mixture models using single-layer estimations. In this approach, the criteria to measure the degree of component overlap have to be identified. Although various concepts have been developed to measure the degree of overlap, such as entropy [5], misclassification rate [4,6], and unimodality [4], they have not been satisfactorily compared yet.

### 2.3. Optimization

Merging components has also been discussed in the scenario of optimizing parameters in the mixture models. Ueda et al. [21] proposed splitting and merging mixture components to obtain better estimations, and Minagawa et al. [22] revised their methods to search the models with higher likelihoods. Zhao et al. [23] considered randomly swapping the mixture components during optimization, which allows a more flexible search than splitting and merging components. Because these methods aim only to optimize the models, there remains the problem of interpreting them.

We also refer to the agglomerative hierarchical clustering as a similar approach to merging components. Our methods are similar to the Bayesian hierarchical clustering methods [24,25] in that the number of merging is automatically decided. However, our approaches can not only create clusters, but also evaluate their shape and closeness under the assumption that the mixture models are given.

### 2.4. Visualization

Methods of interpreting clustering structures have been studied along with visualization methods. Visualizing the values of criteria with a dendrogram is useful for understanding cluster structures among sub-components [6]. Class-preserved projections [26] and parametric embedding [27] were proposed for visualizing structures among upper-clusters by reducing data dimension. We present a method to interpret both structures uniformly based on the MC and NMC.

## 3. Merging Mixture Components

We assume that data $x^{N}=x_{1},\cdots ,x_{N}$ and a finite mixture model are given. The probability distribution of the model *f* is written as follows:

$$f\left(x\right)=\sum_{k=1}^{K}\rho_{k}g_{k}\left(x\right),$$

where *K* denotes the number of components, $\rho_{1},\cdots ,\rho_{K}$ denote the mixture proportions of each component summing up to one, and $g\left(x|\theta_{1}\right),\cdots ,g\left(x|\theta_{K}\right)$ denote the probability distributions. We assume that the data $x^{N}$ are independently sampled from *f*. The random variable *X* following *f* is called an *observed variable*, because it can be observed as a data point. We also define the *latent variable* Z∈Z{1,⋯,K} as the index of the component from which *X* originated. The pair (X,Z) is called a *complete variable*. The distribution of the latent variable P(Z) and the conditional distribution of the observed variable P(X|Z) can be given by the following:

$$P\left(Z=k\right)=\rho_{k},\;P\left(X|Z=k\right)=g_{k}\left(X\right).$$

In the case that *f* is not known, we will replace *f* by its estimation $\hat{f}$ under the assumption that $\hat{f}$ is so close to *f* that $x^{N}$ can be approximately regarded as samples from $\hat{f}$.

We discuss identifying cluster structures in $x^{N}$ and *f* by merging mixture components as described below. First, we define a criterion function denoted as Crit:Z×Z→R, which measures the degree of overlap or closeness between two components. For simplicity, we change the sign of the original definitions as needed so that Crit takes smaller values as the components are closer. Then, we choose the closest two components that minimize the criterion and merge them. By repeating the merging process several times, we finally obtain clusters. We show the pseudo-code and computational complexity of this procedure in Appendix A.

## 4. Essential Conditions

In this section, we propose three *essential conditions* that the criteria should satisfy, so that the criteria can be compared in terms of the conditions. To establish the conditions, we restrict the criteria to those that can be calculated from the posterior probability of the latent variables ${\left\{\gamma_{k}\left(x_{n}\right)\right\}}_{k,n}$ defined as follows:

$$\gamma_{k}\left(x_{n}\right)P\left(Z=k|X=x_{n}\right)=\frac{\rho_{k}g_{k}\left(x_{n}\right)}{f\left(x_{n}\right)},$$

where *k* is the index of the component. After merging the components *i* and *j*, the posterior probability can be easily updated as follows:

$$\gamma_{i\cup j}\left(x_{n}\right)P\left(Z\in \left\{i,j\right\}|X=x_{n}\right)=\gamma_{i}\left(x_{n}\right)+\gamma_{j}\left(x_{n}\right).$$

Note that some other merging methods reestimate the distribution of the merged components as a single component [4]. We do not consider these in this study because they lack the benefit that the merged components can have complex shapes.

For later use, we define Best(Crit) and Worst(Crit) as the best and worst values that the criteria can take:

$$\begin{array}{cc} \mathrm{Best}(\mathrm{Crit}) & \min\mathrm{Crit}\left(i,j\right)\;w.r.t.\;{\left\{\gamma_{k,n}\right\}}_{k,n},\\ \mathrm{Worst}(\mathrm{Crit}) & \max\mathrm{Crit}\left(i,j\right)\;w.r.t.\;{\left\{\gamma_{k,n}\right\}}_{k,n}, \end{array}$$

where ${\left\{\gamma_{k,n}\right\}}_{k,n}$ is a set of K×N real values in [0,1] that satisfies $\sum_{k}\gamma_{k,n}=1$ for all *n*.

We formulate the three conditions. They provide natural and minimum conditions on the behaviors in the extreme cases that the components are entirely overlapped or separated and on the scale invariance of the criteria. The conditions for the moderate cases that the components partially overlap should be investigated in further studies.

First, we define the condition that a criterion should take the best value when the two components entirely overlap. It is formally defined as follows.

**Definition** **1.** *If a criterion satisfies that*

$$\left(\forall n,\;g_{i}\left(x_{n}\right)=g_{j}\left(x_{n}\right)\right)\Rightarrow \mathrm{Crit}\left(i,j\right)=\mathrm{Best}\left(\mathrm{Crit}\right),$$

*then, we say that it satisfies the condition BO* (best in entirely overlap).

Next, we define the condition that the criterion should take the worst value when the two components are entirely separated.

**Definition** **2.** *We consider that the sequence of the models ${\left\{f_{t}=\sum_{k}\rho_{k,t}g_{k,t}\right\}}_{t=1}^{\infty }$ satisfies the following:*

(1) $$\forall n,\;g_{i,t}\left(x_{n}\right)g_{j,t}\left(x_{n}\right)\to 0$$

*as t→∞. We define ${\mathrm{Crit}}_{t}\left(i,j\right)$ as the criterion value based on $f_{t}$. Then, if (1) implies that*

$$\lim_{t\to \infty }{\mathrm{Crit}}_{t}\left(i,j\right)\to \mathrm{Worst}\left(\mathrm{Crit}\right),$$

*we say that it satisfies the condition WS* (worst in entirely separate).

Note that this definition is written using limits in case that the distribution of the components has support in the entire space, such as the Gaussian distributions.

Finally, we define the condition that the value of the criterion should be invariant with the scale of mixture proportions.

**Definition** **3.** *We consider that the components i and j are isolated from the other components, i.e., the sequence of the models ${\left\{f_{t}=\sum_{k}\rho_{k,t}g_{k,t}\right\}}_{t=1}^{\infty }$ satisfies the following:*

$$\left(g_{i,t}\left(x_{n}\right)+g_{j,t}\left(x_{n}\right)\right)g_{k,t}\left(x_{n}\right)\to 0$$

*for all k≠i,j and n as t→∞. In addition, we consider another sequence of the mixture model ${\left\{{\bar{f}}_{t}=\sum_{k}{\bar{\rho }}_{k,t}g_{k,t}\right\}}_{t=1}^{\infty }$ with different scales on the mixture proportions of the components i and j, i.e., ${\bar{\rho }}_{k,t}=a\rho_{k,t}\;\left(k=i,j\right)$ holds for some a>0. We define ${\bar{\mathrm{Crit}}}_{t}\left(i,j\right)$ as the criterion value based on ${\bar{f}}^{\left(t\right)}$. Then, we say that the criterion satisfies the condition SI* (Scale invariance) *if for any a, the following holds*:

$$\lim_{t\to \infty }{\mathrm{Crit}}_{t}\left(i,j\right)=\lim_{t\to \infty }{\bar{\mathrm{Crit}}}_{t}\left(i,j\right).$$

## 5. Modifying Merging Methods

In this section, we introduce the existing merging criteria and propose new criteria by modifying them so that they can satisfy more essential conditions.

### 5.1. Entropy-Based Criterion

First, we introduce the *entropy-based criterion* (Ent) proposed by Baudry et al. [5]. It selects the components that reduce the entropy of the latent variable the most. This criterion, denoted as ${\mathrm{Crit}}_{\mathrm{Ent}}$, is formulated as follows:

$$-{\mathrm{Crit}}_{\mathrm{Ent}}\left(i,j\right)\sum_{n=1}^{N}\left(\Psi \left(\gamma_{i}\left(x_{n}\right)\right)+\Psi \left(\gamma_{j}\left(x_{n}\right)\right)-\Psi \left(\gamma_{i\cup j}\left(x_{n}\right)\right)\right),$$

where Ψ(x)−xlogx.

However, it violates the conditions BO and SI. Therefore, we propose to modify it in two regards. First, we correct the scale of the weights to make ${\mathrm{Crit}}_{\mathrm{Ent}}$ satisfy SI. We propose a new criterion ${\mathrm{Crit}}_{\mathrm{NEnt}}1$ defined as follows:

$$-{\mathrm{Crit}}_{\mathrm{NEnt}}1\left(i,j\right)\frac{-{\mathrm{Crit}}_{\mathrm{Ent}}\left(i,j\right)}{N\left({\tilde{\rho }}_{i}+{\tilde{\rho }}_{j}\right)},$$

where ${\tilde{\rho }}_{k}\sum_{n}\gamma_{k}\left(x_{n}\right)/N$. This satisfies the condition SI.

Next, we propose removing the effects of the weight biases to make ${\mathrm{Crit}}_{\mathrm{NEnt}}1$ satisfy BO. We further introduce a new criterion ${\mathrm{Crit}}_{\mathrm{NEnt}}2$ defined as follows:

$$\begin{array}{cc} {\mathrm{Crit}}_{\mathrm{NEnt}}2\left(i,j\right)\; & \frac{{\mathrm{Crit}}_{\mathrm{NEnt}}1\left(i,j\right)}{{\tilde{H}}_{i,j}\left(Z\right)},\\ {\tilde{H}}_{i,j}\left(Z\right)\; & \sum_{k\in \{i,j\}}\Psi \left(\frac{{\tilde{\rho }}_{k}}{{\tilde{\rho }}_{i}+{\tilde{\rho }}_{j}}\right). \end{array}$$

This satisfies all conditions: BO, WS, and SI.

### 5.2. Directly Estimated Misclassification Probabilities

Second, we introduce the criterion named directly estimated misclassification probabilities (DEMP) [4]. It selects the components with the highest misclassification probabilities. The criterion is formulated as follows:

$$-{\mathrm{Crit}}_{\mathrm{DEMP}}\left(i,j\right)\max\left\{{\tilde{M}}_{j,i},{\tilde{M}}_{i,j}\right\},$$

where

$$\begin{array}{c} {\tilde{M}}_{j,i}\tilde{P}\left(\hat{z}\left(X\right)=j|Z=i\right)\frac{\sum_{n}\gamma_{i}\left(x_{n}\right)1\left(\hat{z}\left(x_{n}\right)=j\right)}{N{\tilde{\rho }}_{i}},\\ \hat{z}\left(x\right)=\underset{k=1,\cdots ,K}{\arg\;\max}\gamma_{k}\left(x\right). \end{array}$$

However, this violates the condition BO when $\hat{z}\left(x_{n}\right)$ is not *i* or *j* for some *n*. Therefore, we modify it by restricting the choice of the latent variable to component *i* or *j*. We define ${\hat{z}}_{i,j}\left(x\right)$ as follows:

$${\hat{z}}_{i,j}\left(x\right)\underset{k=i,j}{\arg\;\max}\gamma_{k}\left(x_{n}\right)$$

and define ${\mathrm{Crit}}_{\mathrm{DEMP}}2$ by replacing $\hat{z}\left(x\right)$ with ${\hat{z}}_{i,j}\left(x\right)$ in the definition of ${\mathrm{Crit}}_{\mathrm{DEMP}}$. Then, this satisfies all essential conditions.

### 5.3. Mixture Complexity

**Figure 3:**
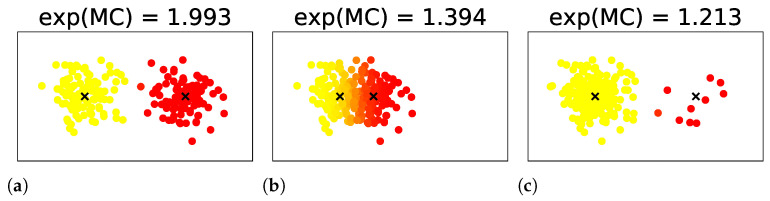
Examples of MC for mixtures of two components. Images are obtained from [7].

Finally, we propose a new criterion based on mixture complexity (MC) [7]. MC is an extended concept of (the logarithm of) the number of clusters into a real value considering the overlap and bias among the components. It is defined based on information theory, and formulated as follows:

$$\mathrm{MC}\left({\left\{\gamma_{k}\left(x_{n}\right)\right\}}_{k,n};{\left\{w_{n}\right\}}_{n}\right)\sum_{k=1}^{K}\Psi \left({\tilde{\rho }}_{k}\right)-\sum_{n=1}^{N}\frac{w_{n}}{W}\sum_{k=1}^{K}\Psi \left(\gamma_{k}\left(x_{n}\right)\right),$$

where ${\left\{w_{n}\right\}}_{n}$ denotes the weights of the data $x^{N}$, $W\sum_{n}w_{n}$ denotes their sum, and ${\tilde{\rho }}_{k}$ is redefined as ${\tilde{\rho }}_{k}\sum_{n}w_{n}\gamma_{k}\left(x_{n}\right)/W$. Examples of MC for mixtures of two components are shown in Figure 3. In them, the exponential of the MCs take values between 1 and 2, according to the uncertainty in the number of clusters induced by the overlap or weight bias between the components.

We first propose a new merging criterion ${\mathrm{Crit}}_{\mathrm{MC}}$ to select the components whose MCs are the smallest. It is defined as follows:

$${\mathrm{Crit}}_{\mathrm{MC}}\left(i,j\right)\mathrm{MC}\left({\left\{\frac{\gamma_{k}\left(x_{n}\right)}{\gamma_{i\cup j}\left(x_{n}\right)}\right\}}_{k\in \{i,j\},n};{\left\{\gamma_{i\cup j}\left(x_{n}\right)\right\}}_{n}\right).$$

However, this does not satisfy the condition WS because of the effects of the weight biases. Therefore, we modify it by removing the biases to propose a new criterion, which we call the *normalized mixture complexity* (NMC) ${\mathrm{Crit}}_{\mathrm{NMC}}$. The criterion is defined as follows:

$${\mathrm{Crit}}_{\mathrm{NMC}}\left(i,j\right)\frac{{\mathrm{Crit}}_{\mathrm{MC}}\left(i,j\right)}{{\tilde{H}}_{i,j}\left(Z\right)}.$$

It satisfies all conditions BO, WS, and SI. Note that it is equivalent to ${\mathrm{Crit}}_{\mathrm{NEnt}}2$ because ${\mathrm{Crit}}_{\mathrm{NMC}}=1+{\mathrm{Crit}}_{\mathrm{NEnt}}2$.

We summarize the relationships between the criteria and the essential conditions in Table 1. The modification led to the fulfillment of many conditions.

## 6. Stopping Condition

We also propose a new stopping condition based on NMC. First, we calculate the NMC for the (unmerged) mixture model *f* defined as follows:

$${\mathrm{NMC}}_{0}\frac{\mathrm{MC}\left({\left\{\gamma_{k}\left(x_{n}\right)\right\}}_{k,n};{\left\{1\right\}}_{n}\right)}{\tilde{H}\left(Z\right)}.$$

Since it represents the average degree of separation in the components of *f*, it can be used for the stopping condition for merging. Then, before merging components *i* and *j*, we compare ${\mathrm{Crit}}_{\mathrm{NMC}}\left(i,j\right)$ to ${\mathrm{NML}}_{0}$. If ${\mathrm{Crit}}_{\mathrm{NMC}}\left(i,j\right)\geq {\mathrm{NML}}_{0}$, then the merging algorithm halts without merging components *i* and *j*. Otherwise, the algorithm merges components *i* and *j* and continues further.

Note that this stopping criterion can be applied when a criterion other than ${\mathrm{Crit}}_{\mathrm{NMC}}$ is used. In this case, we use the criterion to search the two closest components and use NMC to decide whether to merge them.

## 7. Clustering Summarization

In this section, we propose methods to quantitatively explain the merging results, using the MC and NMC.

We consider that a mixture model with *K*-component is merged into *L* upper- components. We define the sets $I_{1},\cdots ,I_{L}$ that partition {1,⋯,K} as the sets of the indices that are contained in each upper-component. Then, the MC and NMC among the upper-components, denoted as MC(up) and NMC(up), respectively, can be calculated as follows:

$$\begin{array}{cc} \mathrm{MC}(\mathrm{up})\; & \mathrm{MC}\left({\left\{\sum_{k\in I_{l}}\gamma_{k}\left(x_{n}\right)\right\}}_{l,n},{\left\{1\right\}}_{n}\right),\\ \mathrm{NMC}(\mathrm{up})\; & \frac{\mathrm{MC}(\mathrm{up})}{\sum_{l}\Psi \left({\tilde{\tau }}_{l}\right)}, \end{array}$$

where ${\tilde{\tau }}_{l}$ denotes the weight of the upper-component *l* calculated as follows:

$${\tilde{\tau }}_{l}\frac{1}{N}\sum_{k\in I_{l}}\gamma_{k}\left(x_{n}\right)=\sum_{k\in I_{l}}{\tilde{\rho }}_{k}.$$

For each *l*, the MC and NMC in the sub-components within the upper-component *l*, written as MC(l) and NMC(l), respectively, can be calculated as follows:

$$\begin{array}{cc} \mathrm{MC}(l)\; & \mathrm{MC}\left({\left\{\frac{\gamma_{k}\left(x_{n}\right)}{\sum_{k^{\prime }\in I_{l}}\gamma_{k^{\prime }}\left(x_{n}\right)}\right\}}_{k\in I_{l},n};{\left\{\sum_{k^{\prime }\in I_{l}}\gamma_{k}\left(x_{n}\right)\right\}}_{n}\right),\\ \mathrm{NMC}(l)\; & \frac{\mathrm{MC}(l)}{\sum_{k\in I_{l}}\Psi \left({\tilde{\rho }}_{l}^{\left(k\right)}\right)}, \end{array}$$

where ${\tilde{\rho }}_{l}^{\left(k\right)}$ denotes the relative weight of the sub-component $k\in I_{l}$ calculated as ${\tilde{\rho }}_{l}^{\left(k\right)}{\tilde{\rho }}_{k}/\sum_{k^{\prime }\in I_{l}}{\tilde{\rho }}_{k^{\prime }}.$ NMC is undefined if the denominator is 0.

MC and NMC quantify the degree to which the components are regarded as clusters in different ways: larger values indicate that the components definitely look like different clusters. MC quantifies this by measuring (the logarithm of) the number of clusters continuously, considering the ambiguity induced by the overlap and weight bias among the components. It takes a value between 0 and the logarithm of the number of the components. In contrast, NMC measures the scattering of the components based only on their overlap. It takes a value between 0 and 1. They have also the desirable properties that they are scale invariant and can quantify overlaps among more than two components.

Therefore, we propose the summarization of clustering structures by listing MC(up), NMC(up), component weights, MC(l), and NMC(l) in a table, which we call the *clustering summarization*. The clustering summarization is useful for evaluating the confidence level of the clustering results.

**Figure 4:**
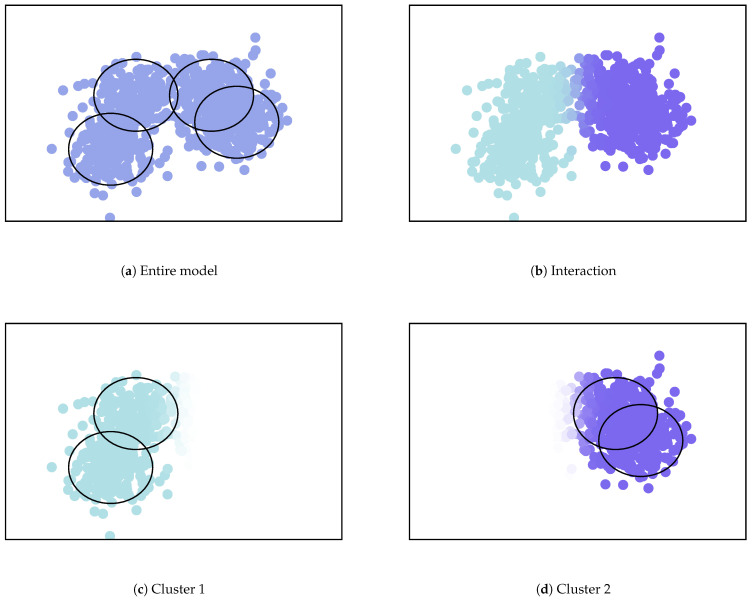
Example of the merged mixture model. Images are obtained from [7].

We show an example of the clustering summarization using the mixture model illustrated in Figure 4. In this example, there are four Gaussian components as illustrated in Figure 4a, and two merged clusters on the left and right sides as illustrated in Figure 4b–d. The clustering summarization is presented in Table 2. For the upper-components, the exponential of MC is almost two, and the NMC is almost one. This indicates that two upper-components can be definitely regarded as different clusters. For both sub-components, the exponential of MC is larger than one. This indicates that they have more complex shapes than a single component. Moreover, the structures within Component 1 are more complex than those in 2, because the MC and NMC are larger.

## 8. Experiments

In this section, we present the experimental results to demonstrate the effectiveness of merging the mixture components and modifying the criteria.

### 8.1. Analysis of Artificial Dataset

To reveal the differences among the criteria, we conducted experiments with artificially generated Gaussian mixture models. First, we randomly created a two-dimensional Gaussian mixture model $f=\sum_{k=1}^{K}\rho_{k}\;N\left(x;\mu_{k},\Sigma_{k}\right)$ as follows:

$$\begin{array}{cc} K\; & 50,\\ \left(\rho_{1},\cdots ,\rho_{K}\right)\; & ∼\mathrm{Dir}(1,\cdots ,1),\\ \mu_{1},\cdots ,\mu_{K}\; & \overset{i.i.d.}{∼}N\left(\mu ;\left[0,0\right],3^{2}\times I_{2}\right),\\ a_{1},b_{1},\cdots ,a_{K},b_{K}\; & \overset{i.i.d.}{∼}U\left[0.5,1.5\right],\\ \Sigma_{k}\; & \left[\left[a_{k},0\right],\left[0,b_{k}\right]\right]\;\left(k=1,\cdots ,K\right), \end{array}$$

where Dir(α,⋯,α) denotes the Dirichlet distribution, and U[m,M] denotes the uniform distribution from *m* to *M*. Then, we sampled 5000 points $x^{5000}$ from *f*, and ran the merging algorithms without stopping conditions. The algorithms were evaluated using the (maximum) intra-cluster distance $D_{\mathrm{intra}}$ and (minimum) inter-cluster distance $D_{\mathrm{inter}}$ defined as follows:

$$\begin{array}{cc} D_{\mathrm{intra}} & \;\max_{k=1,\cdots ,K}\frac{\sum_{n}\gamma_{k}\left(x_{n}\right){∥x_{n}-{\tilde{\mu }}_{k}∥}^{2}}{\sum_{n^{\prime }}\gamma_{k}\left(x_{n^{\prime }}\right)},\\ D_{\mathrm{inter}} & \;\min_{1\leq i<j\leq K}{∥{\tilde{\mu }}_{i}-{\tilde{\mu }}_{j}∥}^{2}, \end{array}$$

where ${\tilde{\mu }}_{1},\cdots ,{\tilde{\mu }}_{K}$ denote the centers of the components defined as

$${\tilde{\mu }}_{k}\frac{\sum_{n}\gamma_{k}\left(x_{n}\right)x_{n}}{\sum_{n^{\prime }}\gamma_{k}\left(x_{n^{\prime }}\right)}.$$

The clustering structure is said to be *better*, as $D_{\mathrm{intra}}$ is smaller and $D_{\mathrm{inter}}$ is larger. Both distances are measured with several *K* and compared among the algorithms with different criteria. Although we may obtain better results for these metrics by using them as merging criteria in a similar way as used in hierarchical clustering [28,29], we used them only for comparison rather than optimizing them.

The experiments were performed 100 times by randomly generating *f* and the data. Accordingly, the ranking of the criteria was calculated for each distance. Table 3 presents the average rank of each criterion. As seen from the table, the modifications of the criteria improved the rank. In addition, DEMP2 and NMC, satisfying all essential conditions, were always in the top three. These results indicate the effectiveness of the essential conditions.

**Figure 5:**
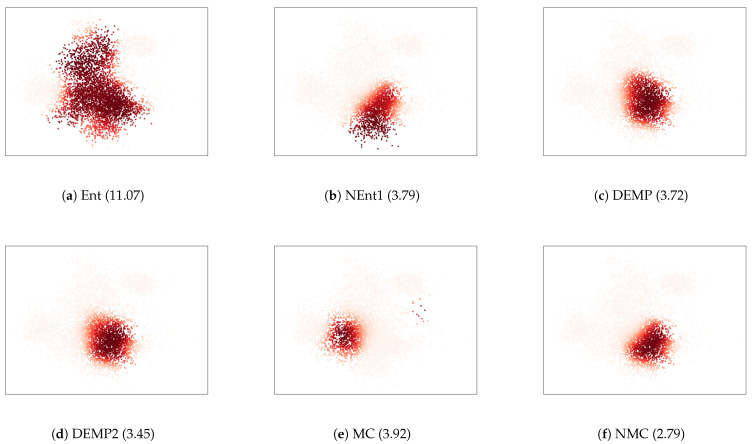
Scatter plots for the cluster with K=20 whose intra-cluster distance is the largest. The thickness of the color corresponds to the posterior probabilities. The numbers in the parenthesis show $D_{\mathrm{intra}}$.

To further investigate the relationships between the essential conditions and resulting cluster structures, we illustrated the cluster obtained in a trial where the intra-cluster distance was the largest in Figure 5. For the criterion Ent, one cluster continued to grow. This is because Ent lacks the condition SI, and is advantageous for larger clusters. For the criterion NEnt1, the growth of the larger clusters was mitigated by adding the condition SI to Ent. Nevertheless, the intra-cluster distances were still large because NEnt lacked the condition BO. It tended to create unnecessarily large clusters because it tended to merge larger and more distant components rather than smaller and closer components. The criterion NMC improved such a disadvantage by adding the condition BO to NEnt1. For the criterion MC, distant components were merged, as the condition WS was not satisfied. NMC overcame this by adding the condition WS to MC. The differences between DEMP and DEMP2 were unclear in Figure 5c,d, and both criteria elucidated the cluster structure well because they satisfied relatively many conditions. We conclude that the essential conditions are effective for obtaining better cluster structures.

### 8.2. Analysis of Real Dataset

We discuss the results of applying the merging algorithms and clustering summarization to eight types of real datasets with true cluster labels. The details of the datasets and processing are described in Appendix B.

#### 8.2.1. Evaluation of Clustering Using True Labels

First, we compared the clustering performance of the merging algorithms by measuring similarity between estimated and true cluster labels. Formally, given the dataset ${\left\{x_{n}\right\}}_{n}$ and the true labels ${\left\{z_{n}^{★}\right\}}_{n}$, we first estimated the clustering structures using ${\left\{x_{n}\right\}}_{n}$ without seeing ${\left\{z_{n}^{★}\right\}}_{n}$, and obtained the estimated labels ${\left\{{\hat{z}}_{n}\right\}}_{n}$. We define $K^{★}$ and $\hat{K}$ as the number of the true and estimated clusters. Then, we evaluated the similarity between ${\left\{z_{n}^{★}\right\}}_{n}$ and ${\left\{{\hat{z}}_{n}\right\}}_{n}$ using the adjusted Rand index (ARI) [30] and F-measure. ARI takes values between -1 and 1, and F-measure takes values between 0 and 1. Their larger value corresponds to better clustering. Both indices can be applied when the number of true and estimated clusters is different.

To run the merging algorithms, the mixture models should be estimated first. In our experiments, we estimated them by the variational Bayes Gaussian mixture model with K=20 [31] implemented in the Scikit-learn package [32]; we adopted this, as it exhibited good performance in our experiments. We used the prior distributions of the mixture proportions as the Dirichlet distributions with α=0.1, and we set the other parameters for prior distributions as the default values in the package. For each dataset, we fitted the algorithm ten times with different initializations and used the best one.

We compared the merging algorithms with three types of model-based clustering algorithms based on the Gaussian mixture model, which are summarized in Table 4. First, we estimated the number of components, using BIC [33]. It selects a suitable model for describing the densities, and the mixture components tend to overlap. Nevertheless, it has been widely used for clustering by regarding each component as a cluster. Second, we estimated the number of clusters using DNML [11,12]. It selects a model whose components can be regarded as clusters by considering the description length of the latent and observed variables. Finally, we estimated the clusters as the mixture of Gaussian mixture models implemented by Malsiher-Walli et al, [20]. By fixing two integers *K* and *L*, *K* Gaussian mixture models were estimated with *L* components. The number of clusters was automatically adjusted by shrinking the redundant clusters. As in the original paper, we set K=30,L=15 (and some specific parameters in the paper) for the DLB dataset and K=10,L=5 for the other datasets.

We estimated the models ten times and compared the average score among the methods. The average number of clusters are listed in Table 5, and F-measure and ARI are listed in Table 6 and Table 7.

Two clusters that achieved the best score and that were obtained by the heuristics proposed in Section 6 are described. The best scores of the merging algorithms exceeded those of all other methods for six out of eight datasets. In particular, the merging methods satisfying many essential conditions, such as DEMP, DEMP2, and NMC, obtained high scores with a smaller number of clusters. Therefore, it can be said that the merging algorithms with more essential conditions are effective for elucidating the clustering structures. Moreover, the scores with NMC-based stopping conditions exceeded those of all other methods for four out of eight datasets.

**Figure 6:**
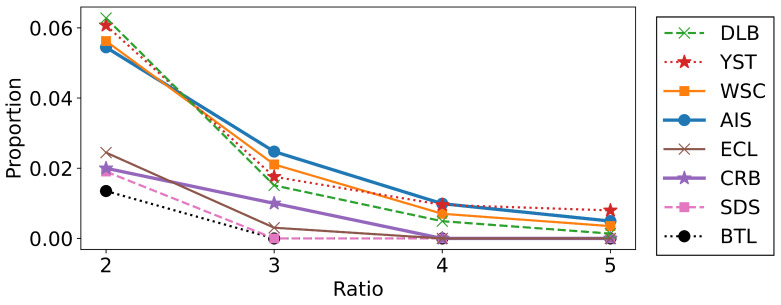
The proportions of the data $x_{n}$ that satisfy $D_{\mathrm{nn}}^{\left(5\right)}\left(x_{n}\right)/\left[\left(1/N\right)\sum_{n^{\prime }}D_{\mathrm{nn}}^{\left(5\right)}\left(x_{n^{\prime }}\right)\right]>$ 2.0, 3.0, 4.0, 5.0.

To further investigate the relationships between the performances of the algorithms and the shapes of the datasets, we estimated the proportion of outliers based on the *k*-nearest neighbor distances $D_{\mathrm{nn}}^{\left(5\right)}$. We calculated the ratio of the 5-nearest neighbor distance $D_{\mathrm{nn}}^{\left(5\right)}\left(x_{n}\right)$ and its average $\left(1/N\right)\sum_{n^{\prime }}D_{\mathrm{nn}}^{\left(k\right)}\left(x_{n^{\prime }}\right)$ for each data point, and we plotted the proportions for which the ratio exceeded 2.0, 3.0, 4.0, and 5.0 in Figure 6. As seen from the figure, the datasets where the merging methods did not work well, such as AIS, DLB, WSC, and YST, contained relatively many outliers. This is reasonable because the merging algorithms do not aim to merge distant clusters. We can conclude that the merging methods are particularly effective when the datasets have fewer outliers or when we want to find the aggregated clusters.

#### 8.2.2. Results of Clustering Summarization

**Figure 7:**
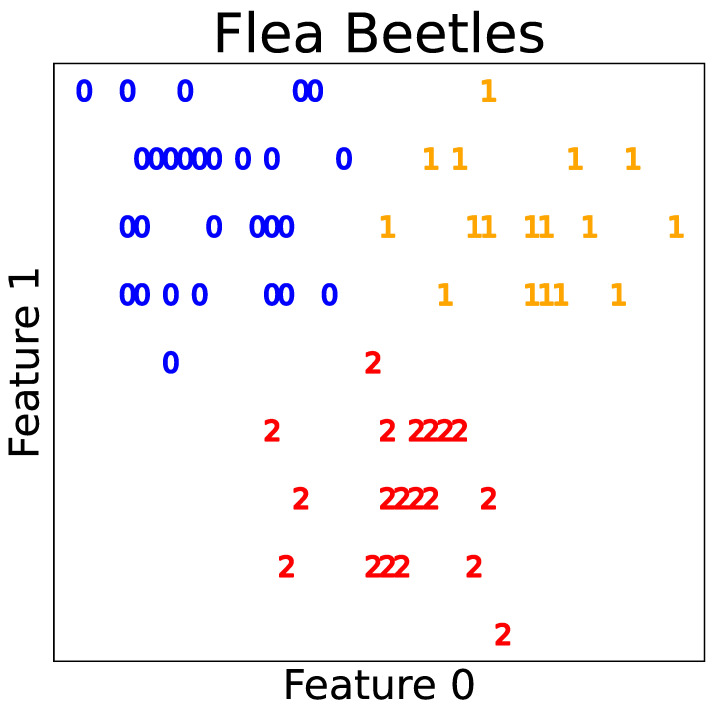
Predicted cluster labels for the Flea beetles dataset.

**Figure 8:**
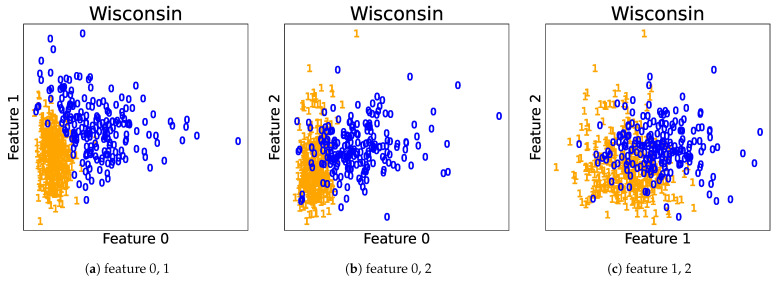
Predicted cluster labels for the Wisconsin breast cancer dataset.

Next, we analyzed the results of the merging methods using the clustering summarization proposed in Section 7. As examples, we show one result obtained using the NMC and NMC-based stopping conditions for the Flea beetles and Wisconsin breast cancer datasets. The clustering results are summarized in Table 8 and Table 9, respectively. For the upper-components in Flea beetles dataset, the exponential of MC(up) was close to 3.0, and NMC(up) was close to 1.0; we see that the effective number of clusters was around three, and the clusters were well-separated. Components 2 and 3 were unmerged, and the exponentials of MC and NMC of Component 1 were close to 1.0 and 0.0, respectively. This indicates that each cluster can be represented by almost a single Gaussian distribution. Furthermore, the (exponentials of) MC and the NMC of the upper-components in the Wisconsin cancer dataset were 1.66 and 0.763, respectively. It can be expected that the situation was a partial overlap of the two clusters. For Components 1 and 2, NMCs were relatively large. This shows that partially separated components are needed to describe each component. MC of Component 2 was smaller than that of Component 1. Then, it is expected that Component 2 had simpler shapes than Component 1; however, the former seemed to have small components that might be outliers because NMC was larger. Plots of the predicted clusters are illustrated for the Flea beetles and Wisconsin breast cancer datasets in Figure 7 and Figure 8, respectively. We observe that the predictions described previously match to the actual plots. Therefore, we can reveal significant information about the clustering structures by observing the clustering summarizations.

#### 8.2.3. Relationships between Clustering Summarization and Clustering Quality

**Figure 9:**
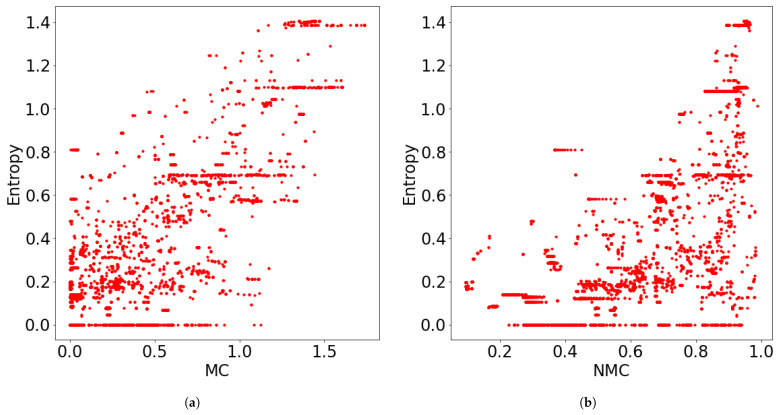
Scatter plots of the MC/NMC and the entropy of the true cluster label.

Finally, we confirmed that MC and NMC in the sub-components were also related to the quality of classification. To confirm this, we conducted additional experiments discussed below. First, we ran the merging algorithms until K=1 without the stopping conditions. Then, for every merged clusters created at $K=K_{\mathrm{start}},\cdots ,1$, we counted the number of data points classified into them. We define $N_{C}^{\left(k\right)}$ as the number of points with true labels *k* classified into the merged cluster *C*. Then, we evaluated the quality of the cluster *C* using the entropy calculated as follows:

$$H_{C}=-\sum_{k=1}^{K^{★}}\frac{N_{C}^{\left(k\right)}}{\sum_{k^{\prime }}N_{C}^{\left(k\right)}}\log\frac{N_{C}^{\left(k\right)}}{\sum_{k^{\prime }}N_{C}^{\left(k\right)}},$$

where the cluster *C* for $\sum_{k^{\prime }}N_{C}^{\left(k\right)}=0$ were omitted. This takes values between 0 and $\log K^{★}$. Smaller values are preferred, because $H_{C}$ becomes small when most of the points within the component share the same cluster label. We calculated the MC/NMC and $H_{C}$ within the clusters for all datasets and merging algorithms, and we plotted the relationships between them in Figure 9. Note that the unmerged clusters were omitted because the NMC could not be defined. From the figure, it is evident that both MC and NMC had positive correlations with $H_{C}$. The correlation coefficients were 0.794 and 0.637 for MC and NMC, respectively. This observation is useful in applications. If the obtained cluster has smaller MC and NMC, then we can confirm that it contains only one group. Otherwise, we need to assume that it contains more than one group. Therefore, we conclude that MC and NMC indicate the confidence level of the cluster structures.

## 9. Discussion

To improve the interpretability of the mixture models with overlap, we have established novel methodologies to merge the components and summarize the results.

For merging mixture components, we proposed essential conditions that the merging criteria should satisfy. Although there have been studies creating some rules in the clustering approach [34,35], they have not been applied to clustering by merging components. The proposed essential conditions for merging criteria contributed to comparing and modifying existing criteria. The limitation of our conditions is that they only provide the necessary conditions for extreme cases, where the components are entirely overlapped or separated. The conditions for the moderate cases that the components partially overlap should be investigated in further studies.

We also proposed a novel methodology to interpret the merging results based on clustering summarization. While previous studies [6,26,27] have focused on interpreting the structures among sub-components or upper-clusters only, our methods can quantify both structures uniformly based on the MC and NMC. They represented the overview of the structures in the mixture models by evaluating how much the components were distinguished based on the degree of overlap and weight bias.

We verified the effectiveness of our methods, using artificial and real datasets. In the artificial data experiments, we confirmed that the intra- and inter-cluster distances were improved corresponding to the modification of the criteria. Further, by observing the clusters with maximum intra-cluster distance, we found that the essential conditions were helpful to prevent the clusters from merging distant components or growing too much. In the real data experiments, we confirmed that the best scores of the proposed methods were better than the comparison methods for many datasets, and the scores obtained using the stopping condition were also better for the datasets containing relatively smaller outliers. In addition, we confirmed that the clustering summary was helpful to interpret the merging results. It was related to the shape of the clusters, weight biases, and the existence of the outliers. Further, we found that the MC and NMC within the components were also related to the quality of the classification. Therefore, the clustering summary also represented the confidence level of the cluster structures.

## 10. Conclusions

We have established the framework of theoretically interpreting overlapping mixture models by merging the components and summarizing merging results. First, we proposed three essential conditions for evaluating cluster-merging methods. They declared necessary properties that the merging criterion should satisfy. In this framework, we considered Ent, DEMP, and MC and their modifications to investigate whether they satisfied the essential conditions. The stopping condition based on NMC was also proposed.

Moreover, we proposed the clustering summarization based on MC and NMC. They quantify how overlapped the clusters are, how biased the clustering structure is, and how scattered the components are in a respective cluster. We can conduct this analysis from higher level clusters to lower level components to give a comprehensive survey of the global clustering structure. We then quantitatively explained the shape of the clusters, weight biases, and existence of the outliers.

In the experiments, we empirically demonstrated that the modification of the merging criteria improved the ability to find better clustering structures. We also investigated the merging order for each criterion and found that the essential conditions were helpful to prevent the clusters from merging distant components or growing too much. Further, we confirmed, using the real dataset, that the clustering summary revealed varied information in the clustering structure, such as the shape of the clusters, weight biases, the existence of the outliers, and even the confidence level of the cluster structures. We believe that this methodology gives a new view of the interpretability/explainability for model-based clustering.

We have studied how to interpret the overlapping mixture models after they were estimated. It remains for future study to apply merging criteria even in the phase of estimating mixture models.

## Figures and Tables

**Table 1:** Summary of the relationships between the criteria and the essential conditions. Check marks are attached to the conditions that are satisfied.

Before Modification				After Modification			
criterion	BO	WS	SI	criterion	BO	WS	SI
Ent		✓		NEnt1		✓	✓
DEMP	(✓)	✓	✓	DEMP2	✓	✓	✓
MC	✓		✓	NMC = NEnt2	✓	✓	✓

**Table 2:** Example of a clustering summarization.

Upper-Components			
MC (exp):		0.647 (1.91)	
NMC:		0.933	
**Component 1**		**Component 2**	
Weight:	0.494	Weight:	0.506
MC (exp):	0.566 (1.76)	MC (exp):	0.324 (1.38)
NMC:	0.817	NMC:	0.467

**Table 3:** Average ranks of the criteria. For each *K*, the best rank is denoted in boldface.

	$D_{\mathrm{intra}}$				
*K*	40	30	20	10	5
Ent	6.00	6.00	6.00	5.94	5.58
NEnt1	3.67	4.15	3.93	4.15	4.18
DEMP	4.30	4.56	4.07	2.57	2.13
DEMP2	2.40	2.52	2.45	2.12	**1.91**
MC	**2.25**	2.04	3.21	4.53	4.94
NMC	2.37	**1.73**	**1.35**	**1.69**	2.27
	$D_{\mathrm{inter}}$				
*K*	40	30	20	10	5
Ent	5.02	5.14	5.38	5.50	5.19
NEnt1	4.29	4.23	4.26	4.56	3.99
DEMP	4.99	4.97	4.82	2.88	2.63
DEMP2	3.56	3.55	3.17	2.85	2.48
MC	2.09	2.06	2.35	3.90	4.86
NMC	**1.06**	**1.04**	**1.03**	**1.29**	**1.84**

**Table 4:** Overview of the comparison methods.

Abbreviation	Method	Reference
GMM + BIC	GMM and BIC criterion	[33]
GMM + DNML	GMM and DNML criterion	[11,12]
MixMix	Mixture of Gaussian mixture models	[20]

**Table 5:** Estimated number of clusters. Merge (best F-measure) is the number of clusters when F-measure is highest. Merge (best ARI) is the number of clusters when ARI is highest. Merge (NMC) is the number of clusters obtained by the NMC-based stopping condition.

Dataset		AIS	BTL	CRB	DLC	ECL	SDS	WSC	YST
$K^{★}$		2	3	4	4	5	3	2	2
GMM + BIC		3.0	2.6	3.0	6.6	4.0	2.0	3.0	3.5
GMM + DNML		1.0	1.0	1.0	2.8	4.0	1.0	2.0	1.0
MixMix		2.7	1.2	1.0	7.4	4.5	3.2	2.1	2.8
Merge (Best F-measure)	Ent	18.0	20.0	19.1	17.6	19.0	19.4	19.2	18.6
	NEnt1	2.0	3.0	5.7	10.2	8.9	6.7	5.3	8.8
	DEMP	2.0	3.0	4.3	4.9	6.2	4.7	3.4	2.3
	DEMP2	2.0	3.0	4.3	4.9	5.7	4.1	2.7	2.0
	MC	2.0	3.0	5.6	3.6	4.9	7.1	2.7	2.0
	NMC	3.0	3.0	4.3	7.1	6.4	4.3	2.0	2.1
Merge (Best ARI)	Ent	19.0	20.0	19.4	17.6	19.0	19.4	19.2	18.6
	NEnt1	3.0	3.0	5.7	3.2	7.0	6.7	5.3	8.8
	DEMP	2.4	3.0	4.3	4.9	6.2	4.9	3.6	2.3
	DEMP2	2.0	3.0	4.3	5.0	6.1	4.5	2.7	2.0
	MC	2.0	3.0	5.6	3.6	4.9	7.1	4.0	2.0
	NMC	4.0	3.0	4.3	7.0	6.4	4.4	2.0	2.1
Merge (NMC)	Ent	19.0	20.0	17.8	17.6	19.0	18.1	19.2	18.6
	NEnt1	4.0	3.0	3.6	6.9	4.7	4.9	5.6	8.9
	DEMP	4.8	3.0	3.3	9.1	5.7	5.3	4.4	8.0
	DEMP2	5.0	3.0	3.3	9.2	5.7	5.2	3.6	8.5
	MC	5.0	3.0	6.2	11.3	9.0	8.6	6.1	9.7
	NMC	4.0	3.0	3.1	6.4	4.3	3.2	2.9	6.9

**Table 6:** F-measure for the real datasets. For each merging algorithm, scores that exceed all comparison methods are denoted in boldface.

Dataset		AIS	BTL	CRB	DLC	ECL	SDS	WSC	YST
GMM + BIC		0.912	0.805	0.810	0.734	0.787	0.794	0.857	0.864
GMM + DNML		0.671	0.590	0.400	0.903	0.787	0.500	0.914	0.850
MixMix		0.925	0.578	0.400	0.761	0.829	0.849	0.947	0.826
Merge (Best)	Ent	0.916	**0.986**	**0.866**	**0.931**	**0.874**	**0.900**	0.897	**0.867**
	NEnt1	0.901	**0.986**	**0.889**	**0.922**	**0.860**	**0.928**	0.904	**0.868**
	DEMP	0.906	**0.986**	**0.877**	**0.952**	**0.874**	**0.908**	0.905	**0.942**
	DEMP2	0.906	**0.986**	**0.877**	**0.952**	**0.875**	**0.912**	0.905	**0.944**
	MC	**0.931**	**0.986**	**0.863**	**0.921**	**0.870**	**0.886**	0.886	**0.928**
	NMC	0.916	**0.986**	**0.893**	**0.949**	**0.875**	**0.938**	0.945	**0.942**
Merge (NMC)	Ent	0.892	**0.986**	**0.822**	**0.931**	**0.874**	**0.852**	0.897	**0.867**
	NEnt1	0.892	**0.986**	**0.828**	**0.905**	0.823	0.822	0.904	**0.868**
	DEMP	0.822	**0.986**	**0.823**	0.758	**0.867**	**0.886**	0.881	**0.870**
	DEMP2	0.805	**0.986**	**0.822**	0.754	**0.867**	**0.892**	0.880	0.820
	MC	0.803	**0.986**	**0.831**	0.706	**0.860**	**0.878**	0.858	0.771
	NMC	0.892	**0.986**	**0.828**	**0.916**	**0.848**	0.810	0.925	**0.878**

**Table 7:** ARI for the real datasets. For each merging algorithm, scores that exceed all comparison methods are denoted in boldface.

Dataset ($K^{★}$)		AIS	BTL	CRB	DLC	ECL	SDS	WSC	YST
GMM + BIC		0.743	0.603	0.595	0.506	0.590	0.542	0.617	0.516
GMM + DNML		0.000	0.000	0.000	0.870	0.590	0.000	0.685	0.000
MixMix		0.751	0.110	0.000	0.501	0.673	0.623	0.799	0.589
Merge (Best)	Ent	0.700	**0.958**	**0.707**	**0.913**	**0.759**	**0.767**	0.688	0.508
	NEnt1	0.701	**0.958**	**0.739**	0.852	**0.719**	**0.810**	0.688	0.511
	DEMP	0.666	**0.958**	**0.732**	**0.934**	**0.763**	**0.782**	0.734	**0.769**
	DEMP2	0.657	**0.958**	**0.731**	**0.936**	**0.763**	**0.788**	0.732	**0.773**
	MC	0.741	**0.958**	**0.700**	0.849	**0.744**	**0.745**	0.664	**0.709**
	NMC	0.700	**0.958**	**0.748**	**0.928**	**0.769**	**0.832**	0.791	**0.760**
Merge (NMC)	Ent	0.700	**0.958**	**0.626**	**0.913**	**0.759**	**0.657**	0.688	0.508
	NEnt1	0.700	**0.958**	**0.642**	0.834	0.638	0.594	0.688	0.511
	DEMP	0.576	**0.958**	**0.640**	0.523	**0.754**	**0.728**	0.670	0.514
	DEMP2	0.545	**0.958**	**0.639**	0.521	**0.754**	**0.745**	0.670	0.402
	MC	0.534	**0.958**	**0.660**	0.452	**0.700**	**0.728**	0.633	0.313
	NMC	0.700	**0.958**	**0.643**	**0.878**	**0.725**	0.575	0.732	0.524

**Table 8:** Clustering summarization for the Flea beetles dataset.

Upper-Components					
MC (exp):			0.963 (2.62)		
NMC:			0.897		
**Component 1**		**Component 2**		**Component 3**	
Weight:	0.440	Weight:	0.268	Weight:	0.293
MC:	0.057	MC:	0.000	MC:	0.000
(exp)	(1.06)	(exp)	(1.00)	(exp)	(1.00)
NMC:	0.209	NMC:	-	NMC:	-

**Table 9:** Clustering summarization for the Wisconsin breast cancer dataset.

Upper-Components			
MC (exp):		0.509 (1.66)	
NMC:		0.763	
**Component 1**		**Component 2**	
Weight:	0.387	Weight:	0.613
MC (exp):	0.714 (2.04)	MC (exp):	0.270 (1.31)
NMC:	0.613	NMC:	0.676

## Data Availability

All of the datasets used in this paper can be obtained in the manner described in the README file at https://github.com/ShunkiKyoya/summarize_cluster_overlap. Moreover, all of the experimental results can be reproduced by executing the .ipynb files.

## Appendix A. Details of the Merging Algorithm

We show the pseudo-code and computational complexity of the merging algorithm. First, the pseudo-code of merging mixture components is shown in Algorithm A1.

**Algorithm A1** Merging mixture components
**Require**: data *x^N^*, finite mixture model *f*, criterion function Crit.
1: **while** (The number of components) > 1 **do**
2: $i,j:=\underset{i<j}{\arg\;\min}\mathrm{Crit}\left(i,j\right)$
3: **if** a certain stopping condition is satisfied **then**
4: **return** The current components.
5: **end if**
6: Merge components *i* and *j*.
7: **end while**
8: **return** The current components.

Next, we discuss the computational complexity in this algorithm given $x^{N}$ and *f* below. First, the cost of calculating ${\left\{\gamma_{k}\left(x_{n}\right)\right\}}_{k,n}$ can be written as $O(T_{\mathrm{dist}}NK)$, where $T_{\mathrm{dist}}$ is the cost to calculate f(x) for a point. To merge components, it is needed to repeat updating ${\left\{\mathrm{Crit}\left(i,j\right)\right\}}_{i,j}$ and ${\left\{\gamma_{k}\left(x_{n}\right)\right\}}_{k,n}$ at most (K−1) times. The cost for updating ${\left\{\mathrm{Crit}\left(i,j\right)\right\}}_{i,j}$ and ${\left\{\gamma_{k}\left(x_{n}\right)\right\}}_{k,n}$ are $O(T_{\mathrm{crit}}K^{2})$ and O(N), respectively, where $T_{\mathrm{crit}}$ is the cost to calculate Crit(i,j) for a pair of the components. Overall, we need $O(K\left(T_{\mathrm{dist}}+T_{\mathrm{crit}}K^{2}+N\right))$ to complete the algorithm.

For the criteria referred to in this section, their computational complexity $T_{\mathrm{crit}}$ are O(N) for Ent, NEnt1, DEMP2, MC, and NMC (NEnt2), and O(NK) for DEMP.

## Appendix B. Details of the Datasets in the Real Data Experiment

The datasets used in the real data experiment are summarized in Table A1. We show the detail and preprocessing of them below. All variables in the datasets are normalized after they are selected.

**Table A1:** Summary of the real dataset, where *N* denotes the number of points, *d* denotes the number of features, and $K^{★}$ denotes the number of true clusters.

Dataset	Abbreviation	(N,d)	$K^{★}$
AIS	AIS	(202, 3)	2
Flea beatles	BTL	(74, 2)	3
Crabs	CRB	(200, 5)	4
DLBCL	DLB	(7932, 3)	4
Ecoli	ECL	(327, 6)	5
Seeds	SDS	(210, 7)	3
Wisconsin breast cancer	WSC	(569, 3)	2
Yeast	YST	(626, 3)	2

The AIS dataset [36] consists of the physical measurements of athletes who trained at the Australian Institute of Sport. Two cluster labels are male and female. As did Lee and McLachlan [16] and Malsiner-Walli et al. [20], we use three variables: BMI, LBM, and body fat percentage (BFat).

The Flea beetles dataset [37] consists of two physical measurements (width and angle) of flea beetles. Three cluster labels are the different species, named Concinna, Heikertingeri, and Heptapotamica.

The Crabs dataset [38] describes five morphological measurements (frontal lobe size, rear width, carapace length, carapace width, and body depth) of 200 crabs. Four cluster labels are formed by combining two color forms and two sexes (male and female).

The DLBCL dataset [39] contains fluorescent intensities of multiple conjugated antibodies (markers) on the cells derived from the lymph nodes of patients diagnosed with DLBCL (diffuse large B-cell lymphoma). As did Lee and McLachlan [40] and Malsiner-Walli et al. [20], we consider four labels corresponding to the cell populations.

The Ecoli dataset [41,42] contains cellular localization sites of proteins. We consider five variables named mcg, gvh, aac, alm1, and alm2. Binary attributes are omitted here. For the labels, we consider five localization sites named cp, im, imU, om, and pp. The other localization sites are omitted because there are little data assigned to them.

The Yeast dataset [41,42] also describes cellular localization sites of proteins. As did Franczac et al. [43] and Malsiner-Walli et al. [20], we select three variables and two cluster labels from the dataset. For the variables, we consider three attributes of proteins, named mcg, alm, and vac. For the labels, we consider two localization sites named CYT and ME3.

The Seeds dataset [44] consists of the seven geometric parameters of grains: area, perimeter, compactness, length of kernel, width of the kernel, asymmetry coefficient, and length of kernel groove. Three cluster labels are kernels belonging to different varieties of wheat: Kama, Rosa, and Canadian.

The Wisconsin breast cancer dataset [3] describes characteristics of the cell nuclei in the images of breast masses. Two cluster labels are benign and malignant. As did Fraley and Raftery [2] and Malsiner-Walli et al. [20], we select three variables: extreme area, extreme smoothness, and mean texture.
